# A Lateral Flow Assay for Quantitative Detection of Amplified HIV-1 RNA

**DOI:** 10.1371/journal.pone.0045611

**Published:** 2012-09-21

**Authors:** Brittany A. Rohrman, Veronica Leautaud, Elizabeth Molyneux, Rebecca R. Richards-Kortum

**Affiliations:** 1 Department of Bioengineering, Rice University, Houston, Texas, United States of America; 2 Paediatric Department, College of Medicine, Queen Elizabeth Central Hospital, Blantyre, Malawi; Northeastern University, United States of America

## Abstract

Although the accessibility of HIV treatment in developing nations has increased dramatically over the past decade, viral load testing to monitor the response of patients receiving therapy is often unavailable. Existing viral load technologies are often too expensive or resource-intensive for poor settings, and there is no appropriate HIV viral load test currently available at the point-of-care in low resource settings. Here, we present a lateral flow assay that employs gold nanoparticle probes and gold enhancement solution to detect amplified HIV RNA quantitatively. Preliminary results show that, when coupled with nucleic acid sequence based amplification (NASBA), this assay can detect concentrations of HIV RNA that match the clinically relevant range of viral loads found in HIV patients. The lateral flow test is inexpensive, simple and rapid to perform, and requires few resources. Our results suggest that the lateral flow assay may be integrated with amplification and sample preparation technologies to serve as an HIV viral load test for low-resource settings.

## Introduction

Over two-thirds of the 33.3 million people estimated to be infected with HIV worldwide live in the developing world [1]. In response to the HIV/AIDS crisis, access to anti-retroviral therapy (ART) has increased dramatically over the past decade in low- and middle-income countries [2]. However, successful management of HIV requires that patients receiving ART be monitored routinely to assess treatment efficacy and detect treatment failure due to drug resistance. Unfortunately, current laboratory based methods to monitor ART are unaffordable, unavailable, or inappropriate for low-resource settings [3].

Rapid antibody tests are widely available in developing nations, but they cannot be used to monitor HIV progression or treatment efficacy. The standard of care to monitor ART is quantitative viral load testing based on plasma HIV RNA concentration [4]. Although CD4 count has also been used to monitor ART, recent studies suggest that it may not detect early treatment failure adequately [5]. The gold standard method for viral load testing, RT-qPCR, is unsuitable for settings where trained technicians, expensive reagents, electrically powered equipment, and dedicated laboratory space are often unavailable. Therefore, a viral load test that is appropriate for such settings is needed.

A number of commercially available viral load tests have been developed for use at the point-of-care but suffer from drawbacks that limit their widespread implementation [6]–[8]. Many emerging technologies that are better designed for use in developing countries may serve as improved point-of-care viral load tests. A variety of microfluidic systems have been developed to perform nucleic acid amplification on-chip [9]–[11]. However, microfluidic systems often require a syringe pump for operation and additional imaging equipment to acquire results. To avoid the difficulties associated with enzymatic amplification of target RNA, alternative approaches have attempted to improve the sensitivity of nucleic acid detection through signal amplification [12], [13]. Other researchers have developed quantitative tests for p24 antigen [14], [15], which may serve as a surrogate for HIV viral load [7]. Despite these advances, no appropriate point-of-care HIV viral load test is currently available.

Recently, paper-based devices have shown promise as point-of-care diagnostics because paper is inexpensive, portable, disposable by burning, and has the ability to wick fluids by capillarity [16]. The emergence of paper microfluidics has renewed interest in lateral flow tests, which have served as point-of-care tests for decades. For example, recent work has shown that lateral flow tests may achieve a greatly improved limit of detection (LOD) and serve as platforms for multiplexed detection [13], [17], [18]. Still, most examples of paper microfluidic technology are antibody tests or small molecule tests [19]–[22], and much work remains to be done to develop and improve paper-based nucleic acid tests.

Here we present a quantitative lateral flow test for detecting amplified HIV RNA that is appropriate for low-resource settings. Gold nanoparticles conjugated to complementary oligonucleotides are used as probes, and gold enhancement is implemented to improve the LOD. Our results indicate that this lateral flow assay may be used in conjunction with amplification to detect HIV RNA concentrations at clinically meaningful levels.

## Methods

### Lateral Flow Assay (LFA) Development

**Figure 1:**
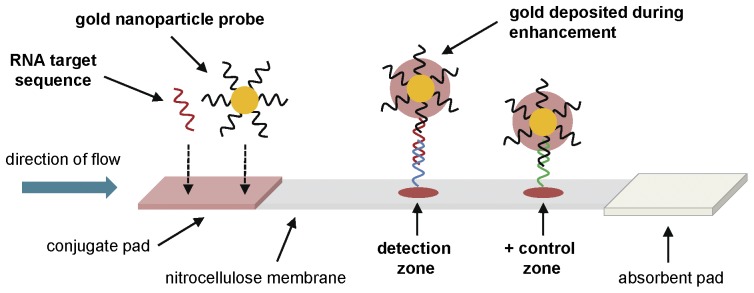
Lateral flow assay design. The lateral flow assay is designed to detect a 142 bp amplified RNA sequence**.** The lateral flow strip consists of a conjugate pad containing gold nanoparticle probes (GNPs), a nitrocellulose membrane containing capture oligonucleotides, and an absorbent pad. Target RNA is dispensed onto the conjugate pad and binds to the GNPs. The target RNA – GNP complex flows down the strip and binds to the target capture sequence, while unbound GNPs bind to the positive control sequence. After wash buffer carries unbound GNPs down the strip, an enhancement solution is added to increase the optical absorbance of the captured GNPs. The signal of the GNPs captured in the detection zone should be proportional to the number of RNA copies dispensed onto the strip, providing quantitative detection.

The LFA was designed to quantitatively detect amplified HIV-1 RNA (Fig. 1). When RNA is dispensed onto the conjugate pad of the strip, the RNA binds to complementary oligonucleotides conjugated to gold nanoparticle probes (GNPs). The target RNA – GNP complex flows down the strip via capillarity and is captured by the target capture sequence in the center of the strip. Unbound GNPs bind to the positive control sequence at the end of the strip. A wash buffer carries unbound GNPs down the strip to decrease the background, while an enhancement solution increases the size and optical absorbance of the bound GNPs. The LFA was designed so that the number of GNPs captured in the detection zone would likely be proportional to the number of RNA copies dispensed onto the strips, providing a quantitative detection modality.

Because the concentration of HIV found in patient plasma samples can be as low as a few copies per milliliter, nucleic acid amplification of genomic HIV RNA must be performed prior to detection with the LFA. The LFA was designed to detect a 142 bp NASBA product amplified from the HIV *gag* gene. To develop and optimize the LFA, we used a 179 bp synthetic RNA sequence containing the NASBA product sequence (Table 1). The synthetic RNA sequence was generated through *in vitro* transcription using the MEGAscript T7 kit (Applied Biosystems) and a plasmid containing the T7 promoter upstream of the template sequence (pIDTBlue, Integrated DNA Technologies). The *in vitro* transcription reaction products were purified using an RNeasy column (QIAGEN, RNeasy Cleanup Protocol) and quantified by measuring absorbance at 260 nm.

**Table 1:** Sequences used for lateral flow assay and NASBA.

Function	Name	Sequence
**Synthetic target sequence for LFA**	IVTX1	5′-GGCGAATTGGGTACGATCGATGCGGCCTCCTCGAGTGCTATGTCACTTCCCCTTGGTTCTCTCATCTGGCCTGGTGCAATAGGCCCTGCATGCACTGGATGCAATCTATCCCATTCTGCAGCTTCCTCATTGATGGTCTCTTTTAACATTTGCATGGCTGCTTGATGTCCCCCCACTG-3′
**Probe oligonucleotide on GNPs**	GNP6B	5′-CAGAATGGGATAGATTGCAT/PEG_18_/AAAAAAAAAAAAAA/SH-3′
**LFA target capture sequence**	CPT1C	5′-GACCATCAATGAGGAAGCTG-3′
**LFA positive control sequence**	POS1	5′-ATGCAATCTATCCCATTCTGTTT-3′
**Forward primer for NASBA**	NASBAp5	5′-*aattctaatacgactcactataggg*CTATGTCACTTCCCCTT-3′
**Reverse primer for NASBA**	NASBAp6	5′-CATCAAGCAGCCATGCAA-3′
**NASBA product sequence**	NBPRD1	5′-TGCTATGTCACTTCCCCTTGGTTCTCTCATCTGGCCTGGTGCAATAGGCCCTGCATGCACTGGATGCAATCTATCCCATTCTGCAGCTTCCTCATTGATGGTCTCTTTTAACATTTGCATGGCTGCTTGATGTCCCCCCACT-3′

### Quantitative Analysis of LFA Performance

For all experiments, the performance of the LFA was assessed quantitatively using image analysis. All LFA strips were scanned (Epson Perfection V500 Photo) and imaged using a stereo microscope (Olympus SZ61) equipped with a color camera (Zeiss, AxioCam MRc5). Images obtained with the stereo microscope were analyzed using a custom Matlab script. The green channel image was cropped to rectangular field-of-view including the captured GNPs and surrounding strip area. An intensity threshold was set by the user to make a mask, which segmented the GNP spot from the background. For images with a high noise level, the mask was drawn manually using the function ‘roispline’ (Mathworks). The intensity of each pixel was subtracted from 255 in order to invert the image data, and the signal-to-background ratio (SBR) was calculated. LFA results are expressed using the SBR.

### LFA Optimization Experiments

The LFA was developed to achieve the best LOD and dynamic range possible while remaining appropriate for low resource settings. We aimed to design a test that costs less than $1, requires an assay time of less than 30 minutes, detects RNA in a volume of less than 50 µL, requires minimal instrumentation, and remains stable for months at ambient temperature. The conditions that were optimized included capture oligonucleotide sequence and concentration, buffer composition, nitrocellulose flow rate, strip width, and gold nanoparticle size (Table 2). To maximize the signal generated by the GNPs, gold and silver enhancement were explored for signal amplification. During gold and silver enhancement, metallic ions are reduced on the surface of the GNPs, increasing their size and optical extinction in order to improve the limit of detection (LOD) of the assay [17], [23].

**Table 2:** Optimized experimental conditions for the lateral flow detection assay.

Experimental condition	Values tested	Optimum value
**Capture oligo concentration**	0.1, 0.2, 0.4, 0.6 mM	0.4 mM
**Type of running buffer**	4X SSC; 4X SSC with 5% formamide; 4X SSC with 5%formamide, 1.4% Triton X-100, and 1% SDS	4X SSX with 5% formamide
**Ratio of detection sequence to random sequence on GNPs**	1∶0, 3∶1, 1∶1, 1∶3	1∶0
**Nitrocellulose flow rate**	75, 135, 180 s/4 cm	135 s/4 cm
**Gold nanoparticle size**	15, 30, 50, 60, 70, 80 nm	50 or 60 nm
**Gold nanoparticle orientation**	5¢ or 3¢ end of probe	3¢ end of probe
**Capture probe length**	16 and 20 bp	20 bp
**Ratio of detection probe to polyA spacer on GNPs**	1∶0, 9∶1, 3∶1, 1∶1, 1∶3	1∶0
**Detection probe sequence**	Sequence adjacent to capture probe, seq. 20 bp away from capture probe	Adjacent sequence
**Ionic strength of wash buffer**	0.1, 0.25, 0.5, 2, 4, 10X SSC	0.5X SSC
**Time to incubate GNP probes and RNA target**	0, 5, 15, 30 min.	0 min.
**Ionic strength of running buffer**	0.5, 1, 2, 4X SSC	4X SSC
**Formamide concentration of wash buffer**	0, 5, 10, 20% formamide	0% formamide
**Lateral flow strip width**	2, 3, 4, 5 mm	2 or 3 mm
**Temperature**	Room temp., 37°C, 42°C, 46°C	37°C

For the optimization experiments, the LFA was performed using a dilution series of *in vitro* transcribed target RNA for each condition to be optimized. The SBR of the detection zone was calculated for each LFA. The SBR was normalized to a maximum value of 100 for each RNA target concentration. Average normalized SBRs for each condition were used to compare conditions. The optimum conditions were defined as those that maximized the SBR, affording the best LOD and largest dynamic range.

### Fabrication of LFA Strips using Optimized Parameters

For all subsequent experiments, LFA strips were fabricated using the optimized parameters. Gold nanoparticles were chosen as probes because of their large optical cross-section and stability [24]. Gold nanoparticle probes (GNPs) were made by conjugating thiolated oligonucleotides (Integrated DNA Technologies) to gold nanoparticles (Ted Pella, NanoXact tannic acid capped gold colloid). The 35 bp oligonucleotide probe sequence contained an 18-atom hexa-polyethyleneglycol internal spacer and 15 bp polyA spacer to maximize loading of oligonucleotides onto the gold nanoparticles (Table 1) [25]. The oligonucleotides (25 µL, 0.1 mM) were incubated for 30 minutes with TCEP-HCl (10 µL, 100 mM) in a total volume of 110 µL to reduce the dithiol bonds. A 600 µL volume of 60 nm gold colloid at the supplied concentration was added to the reaction and incubated overnight on a rotisserie. A 3.57 µL volume of 2% SDS was added to the reaction to reach a final SDS concentration of 0.01%. After 30 minutes of incubation, five 11.57 µL volumes of 2 M NaCl were added to the solution, separated by 2-hour intervals, to reach a total NaCl concentration of 0.15 M. The following day, the GNPs were centrifuged (5000 rcf for 5 minutes) and washed four times with 1 mL of 0.15 M NaCl, 0.01% SDS. The GNPs were resuspended in GNP buffer (0.15 M NaCl, 5% BSA, 0.25% Tween, 10% sucrose) after the final wash.

LFA strips were assembled using glass fiber conjugate pads, nitrocellulose cards, and cellulose absorbent pads. Conjugate pads were cut into 1 cm by 0.5 cm rectangles from glass fiber sheets (GFCP203000, Millipore) using a 60-watt laser cutter (Universal Laser Systems) with 3% power and 5% speed. Ten microliters of GNPs were dispensed onto each pad and dried at room temperature before strip assembly. Absorbent pads (CFSP223000, Millipore) and nitrocellulose strips with a plastic backing (Hi-Flow 135, Millipore) were cut using a guillotine cutter (A-Point Guillotine Cutter Digital Model, Arista Biologicals). Nitrocellulose strips were 3 mm wide, and absorbent pads were cut into 1 cm×0.75 cm rectangles.

Two oligonucleotide sequences were dispensed onto the nitrocellulose strip (0.2 µL volume, 0.4 mM concentration). The target capture sequence, which is complementary to a region of the target RNA sequence, was dispensed in the center of the strip; the positive control sequence, which is complementary to the probe sequence conjugated to the GNPs, was dispensed near the end of the strip (Table 1). After the oligonucleotide solutions dried on the strip at room temperature, strips were exposed to UV light (UVP HL-2000 HybriLinker) at 125 mJ/cm^2^ to crosslink the oligonucleotides to the nitrocellulose. Conjugate and absorbent pads were placed on the adhesive at opposite ends of the nitrocellulose strip, overlapping the nitrocellulose by approximately 2 mm. Strips were used immediately or stored in foil pouches (Clonesaver Resealable Multi Barrier Pouch, Whatman) with desiccant (FTA Card Desiccant Packet, Whatman).

### Characterization of the LFA

After the LFA was optimized, the assay was evaluated for use as a quantitative detection platform. The consistency of LFA performance was tested on two different days. On the first day, one batch of LFA strips was fabricated and the assay was performed in duplicate; on the second day, a second batch of LFA strips was made and the assay was performed in triplicate. Serial dilutions of the *in vitro* transcribed target RNA were used to assess LFA performance. The RNA was prepared in 20 µL of running buffer (4x SSC with 5% formamide) and dispensed onto the conjugate pad of the lateral flow strip. After a 10 minute incubation on a heat block at 37°C, 30 µL of wash buffer (1x SSC) was dispensed onto the conjugate pad and incubated an additional 10 minutes at 37°C. During incubation, gold enhancement solution was prepared by mixing equal volumes of initiator, buffer, activator, and enhancer solutions (Nanoprobes, Gold Enhance LM/Blot). For experiments using silver enhancement, equal volumes of blotting initiator and blotting enhancer were mixed (Ted Pella, Silver Enhancing Kit). Enhancement was performed by adding 30 µL of enhancement solution to the conjugate pad of the strip. LFA strips were left at room temperature to dry. LFA strips were imaged using a flatbed scanner and a stereo microscope and analyzed using the Matlab script as described previously. Dose response curves were constructed to characterize the LFA based on the average signal-to-background ratio calculated for each concentration.

Because the LFA must perform consistently over time in order to be used as a point-of-care test, the stability of the assay was assessed after storing the strips and necessary reagents. At the beginning of the stability study, all lateral flow strips were fabricated on the same day and placed in foil pouches with desiccant. Half of the strips were incubated in an oven at 37°C; half of the strips were kept at room temperature. All buffers and reagents, including gold enhancement reagents, were prepared and set at room temperature for the duration of the study. The lateral flow assay was performed and analyzed on the day of strip creation and 1, 3, 7, 14, 21, and 28 days after strip creation. The two sets of strips tested on the day of fabrication were also used to assess LFA consistency (described in the previous paragraph). Dose response curves for each time point were constructed to assess the performance of the LFA over time.

### Detection of NASBA Products

To demonstrate that the LFA may serve as a detection platform in an HIV-1 viral load assay, NASBA was performed to generate reaction products for detection on LFA strips. A plasmid containing the HIV *gag* sequence (pNL43) served as the template for *in vitro* transcription to generate synthetic copies of HIV *gag* RNA (MEGAscript T7 kit, Applied Biosystems). Zero, 5, 50, 500, and 5000 copies of *gag* RNA served as samples for NASBA reactions.

An additional control of nonspecific, genomic nucleic acid was included in some experiments to demonstrate the specificity of our assay. For this control, total nucleic acid was purified from cultured lymphoblasts (CCRF-CEM cells, American Type Culture Collection) using the QIAamp DNA Blood Mini kit (Qiagen). A total of 740 ng of purified nucleic acid served as a sample for NASBA. To further ensure specificity, we designed the LFA target capture and probe sequences to bind to the amplified sequence between the regions targeted by the NASBA primer sequences. Therefore, any sequence amplified as a result of mispriming should not be detected by the LFA.

In four experiments, NASBA products were generated and detected using the LFA. NASBA was performed using the NucliSens EasyQ Basic Kit as described by the manufacturer (bioMérieux). The KCl concentration in each reaction was 36 mM. Primers NASBAp5 and NASBAp6 as well as the NASBA product sequence are shown in Table 1. Briefly, NASBA reactions were denatured for 4 min. at 65°C, incubated for 4 min. at 41°C, and then allowed to proceed for 90 min. at 41°C after the addition of enzyme. All incubation steps were performed using a heat block. NASBA products consisted of a 142 bp sequence and were diluted by a factor of 10, 100, or 1000 in running buffer. Twenty microliters of diluted products were dispensed onto LFA strips for detection, followed by the wash and enhancement steps as previously described. Dose response curves were made to assess the LFA results.

## Results

### Development and Optimization of the LFA

The LFA was developed and optimized to provide quantitative detection of RNA for eventual use as part of an HIV-1 viral load test. The assay parameters shown in Table 2 were found to optimize performance of the LFA while maintaining conditions that are achievable in low resource settings. The parameters that most affected the LFA performance were capture oligonucleotide concentration, gold nanoparticle size, buffer composition, and temperature. Performing the assay at 37°C on a heat block eliminated any nonspecific binding of GNPs at the detection zone, which is important for avoiding false positive results. Gold and silver enhancement provided signal amplification by increasing the signal-to-background ratio of the detection zone, thereby improving the LOD. Gold enhancement increased the signal-to-background ratio (SBR) by ∼25%, while silver enhancement increased the SBR by ∼15%. LFA strips that underwent silver amplification resulted in a high background, while gold enhancement did not significantly affect the background. Therefore, gold enhancement was chosen for signal amplification in later experiments.

### LFA Performance and Stability

**Figure 2:**
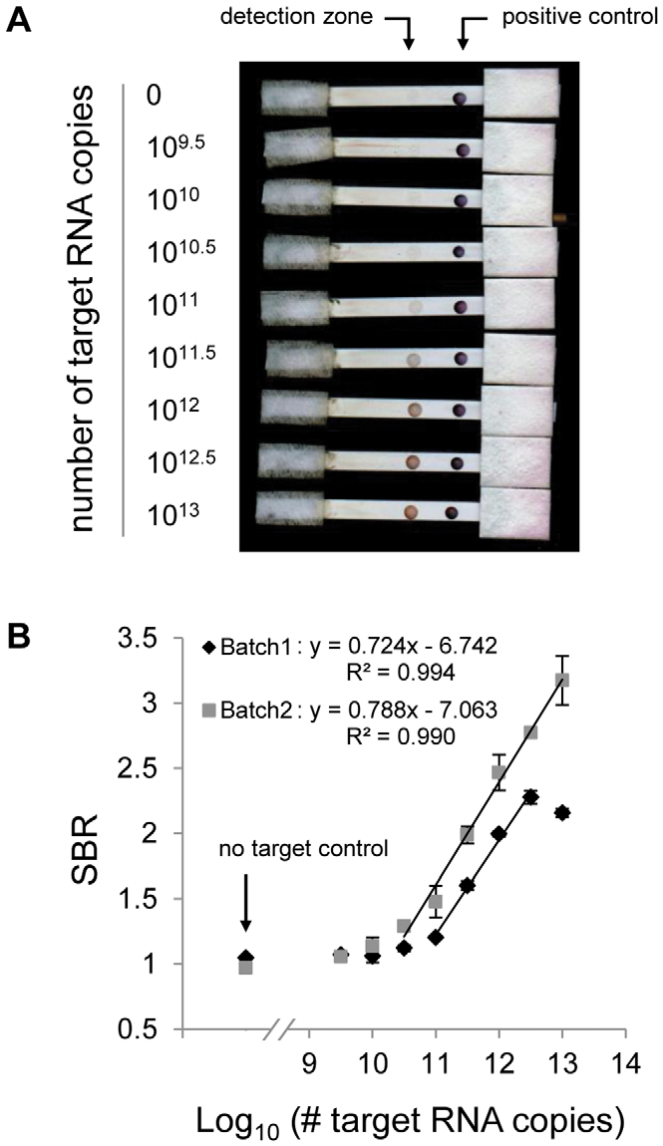
Performance of the optimized lateral flow assay. Lateral flow strips were made and tested on two different days (Batch1 and Batch2). The lateral flow assay was performed in duplicate (Batch1) or in triplicate (Batch2) using a dilution series of *in vitro* transcribed RNA. The number of RNA copies dispensed per strip ranged from 9.5 to 13 log_10_ copies in steps of 0.5 log_10_ copies. (A) Scanned image of one set of lateral flow strips. Note that although the contrast was adjusted in the figure, raw images were used for signal-to-background calculations. (B) Dose response curves based on the average signal-to-background ratio (SBR) for each log_10_ copy number. The negative control SBR is shown for comparison. Error bars represent one standard deviation. The line and regression equation are shown to denote the linear range of the assay.

The performance of the LFA demonstrates that the assay can serve as a quantitative detection platform. Figure 2a shows a scanned image of one set of LFA strips after the assay was performed using various concentrations of target RNA. Figure 2b shows dose response curves for two batches of LFA strips made and tested on different days. The dose response curves are based on the average SBR calculated for each concentration. For Batch 1, the dynamic range extended 2.5 orders of magnitude, from 10.5 to 13 log_10_ RNA copies. The linear region of the dynamic range extended 1.5 orders of magnitude, from 11 to 12.5 log_10_ RNA copies. The LOD, which we define as the first concentration for which the SBR is significantly different from the negative control SBR (p<0.05), was 10.5 log_10_ RNA copies. For Batch 2, the dynamic range extended 3.5 orders of magnitude, from 9.5 to 13 log_10_ RNA copies. The linear region of the dynamic range extended 2.5 orders of magnitude, from 10.5 to 13 log_10_ RNA copies. The LOD was 9.5 log_10_ RNA copies (p<0.05).

The linear range was slightly larger and the LOD was lower for Batch 2 than Batch 1. This difference in performance could be explained by batch-to-batch differences in the preparation of LFA strips, as we have observed some variability in the efficiency of the oligo loading on GNPs (data not shown). Importantly, we did not observe this variability between strips of the same batch. Therefore, a standard curve may be constructed to calibrate the LFA and to account for batch-to-batch performance variability.

**Figure 3:**
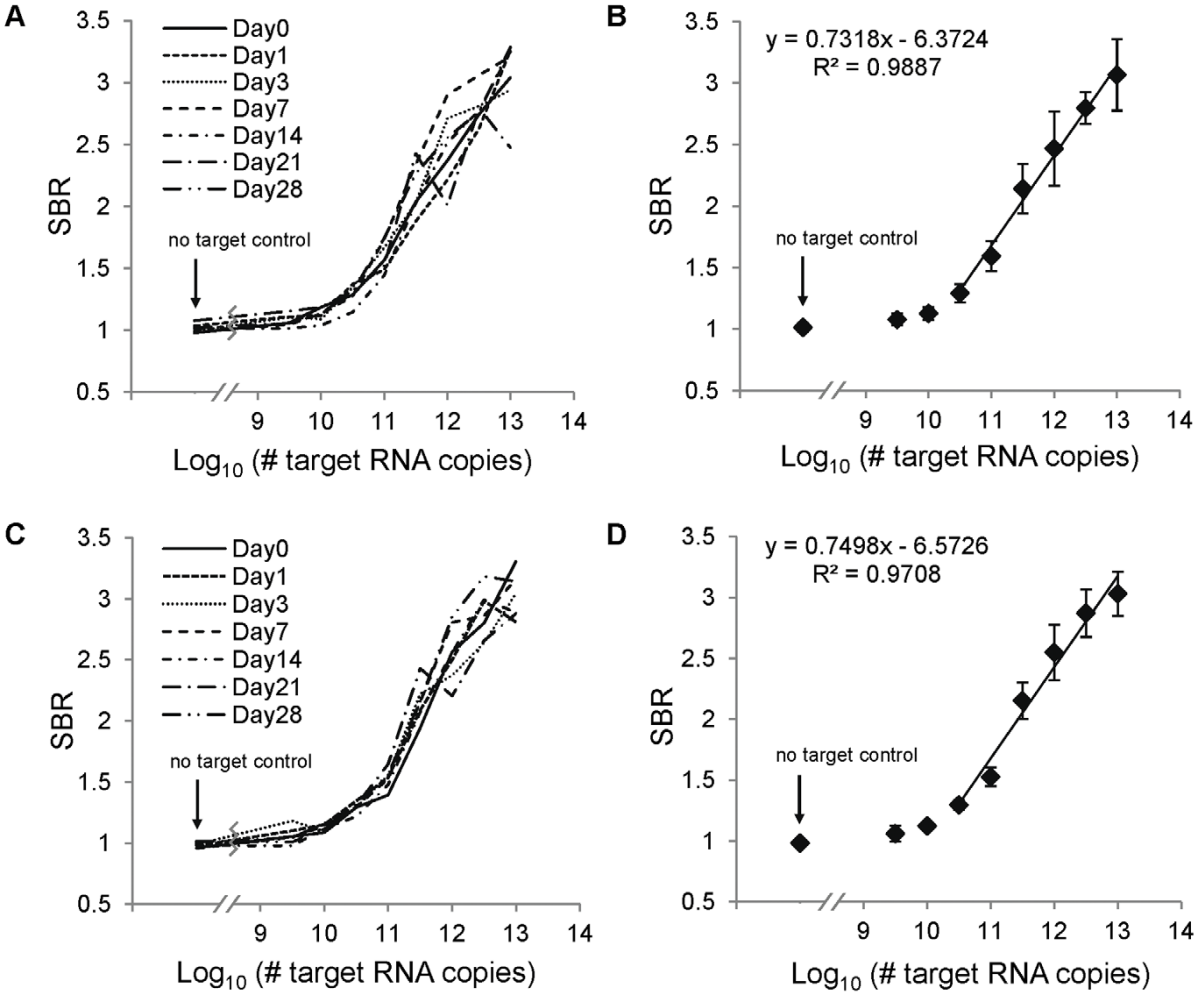
Performance of the lateral flow assay after storage. To assess the effects of storage on LFA performance, lateral flow strips were fabricated on the same day, placed in foil pouches with desiccant, and stored at room temperature or 37°C. The lateral flow assay was performed and analyzed on the day of strip creation and 1, 3, 7, 14, 21, and 28 days after strip creation. The signal-to-background ratio (SBR) for each log_10_ copy number is shown for strips performed on different days. The negative control SBR is shown for comparison. The regression line and equation were calculated for the average SBRs over the linear range of the assay, from 10.5 to 13 log_10_ RNA copies. (A) Dose response curves at each time point and (B) average dose response curve for strips stored at room temperature. (C) Dose response curves at each time point and (D) average dose response curve for strips stored at 37°C.

After storing a single batch of LFA strips for various periods of time at room temperature and 37°C, the performance of the assay remained consistent (Fig. 3). The dose response curves for each day (Fig. 3a and 3c) show that there is no trend in assay performance variability over time. Furthermore, no significant difference was observed between the average performance of strips stored at room temperature (Fig. 3b) and at 37°C (Fig. 3d).Throughout the time course for both storage conditions, the LOD was 9.5 log_10_ RNA copies, and the linear dynamic range extended from 10.5 to 13 log_10_ RNA copies. Average SBRs for each pair of adjacent concentrations were compared using t-tests, which yielded p-values less than 0.05 for all but one pair (p = 0.07 for 12.5 and 13 log_10_ RNA copies for strips stored at 37°C). Thus, the resolution of the assay regardless of storage conditions was 0.5 log_10_ RNA copies. These results demonstrate that the assay performs consistently even after short-term storage, suggesting that the LFA may be used in settings where the storage conditions of test strips may vary greatly.

**Figure 4:**
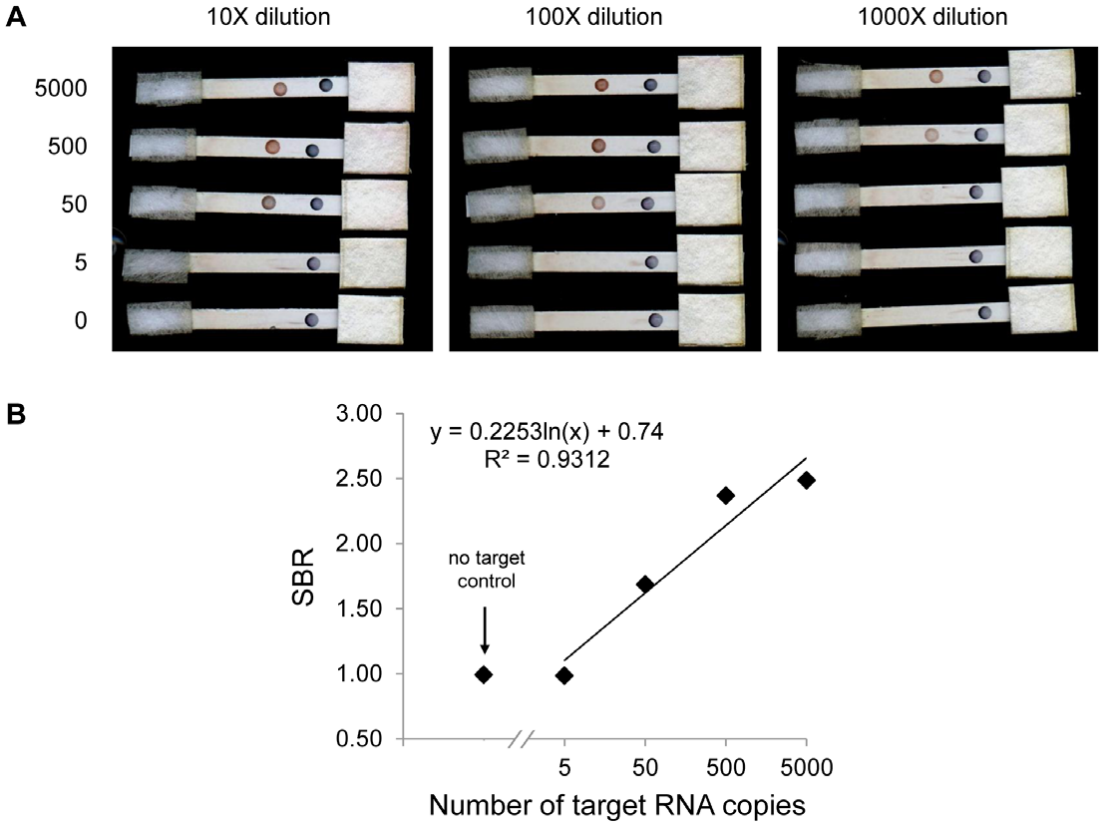
Detection of NASBA products. NASBA was performed using 0, 5, 50, 500, or 5000 copies of *in vitro* transcribed HIV *gag* RNA as a template. Products were diluted by a factor of 10, 100, and 1000, and a 20 µL volume of each dilution was dispensed onto a lateral flow strip for detection. (A) Scanned image of strips for each concentration of NASBA products and dilution factor. Note that although the contrast was adjusted in the figure, raw images were used for signal-to-background calculations. (B) Dose response curve constructed using the signal-to-background ratio of 100-fold diluted products. The negative control SBR is shown for comparison.

### Detection of NASBA Products

NASBA products were detected using the LFA to demonstrate that the LFA can detect amplified target RNA. The upper limit of the clinical range for HIV-1 viral load is about 6 log_10_ copies/mL, while the LFA has an LOD of 9.5 log_10_ RNA copies in 20 µL (equivalent to ∼11 log_10_ copies/mL). Therefore, NASBA was chosen to amplify HIV-1 RNA because NASBA is a well-established isothermal method that provides 10 to 12 orders of magnitude of amplification [26].

The LFA was used to detect NASBA products in four different experiments. Figure 4a shows the LFA strips for one experiment, in which NASBA products generated from 50, 500, and 5000 copies of *gag* RNA were detected by lateral flow strips after dilution by a factor of 10, 100, and 1000 in running buffer. A dilution factor of 100 was sufficient for the SBR to fall within the linear range of the LFA. Figure 4b demonstrates the linear relationship between the SBR and amount of template *gag* RNA (R^2^ = 0.93). The R^2^ values for all four experiments range from 0.90–0.98 with a mean value of 0.94, indicating that the LFA detects amplified RNA quantitatively (Fig. S1). However, the equations of the regression lines differ for each experiment, which suggests that the performance of NASBA varies from day to day. A standard curve should be constructed for each experiment to correct for this variation. The LOD for all four experiments was at least 50 copies, and no signal was present on LFA strips for any of the no-target controls or samples containing background nucleic acid (Fig. S1).Therefore, when combined with NASBA, the LFA may potentially report *gag* RNA concentrations that fall within the clinical range with adequate specificity.

## Discussion

Ideally, HIV viral load is measured before a patient initiates ART and throughout the course of treatment. Typically, patients who begin to experience symptoms have plasma viral load levels exceeding 10,000 viral copies/mL [27]. After ART is initiated, patients are monitored for a significant change in viral load, which is defined as a three-fold or 0.5 log_10_ copies/mL change. After 4–8 weeks, ART is considered to be efficacious if viral load decreases by 1.0 log_10_ copies/mL [28], [29]. Suppression of viral replication is considered successful if the viral load falls below 200 copies/mL [29]. Therapeutic failure due to drug resistance is characterized by a return of low-level viremia, which is defined as a viral load of at least 500–1000 copies/mL [4]. A point-of-care viral load test should achieve an LOD and resolution that accurately identifies these clinical benchmarks.

The lateral flow assay presented here achieves a resolution of 0.5 log_10_ copies/mL over a linear range that extends 2.5 orders of magnitude. When coupled with NASBA, the LFA can detect 50 copies of HIV *gag* RNA. We speculate that this LOD corresponds to a plasma viral load of roughly 1000 copies/mL, assuming that a plasma sample volume of 100 µL will be used and that half of viral RNA from the sample is added to the NASBA reaction. The performance of the LFA when detecting NASBA products suggests that the LFA may be sufficient to detect significant changes in viral load, suppression of viral replication, and therapeutic failure. The LFA only requires a heat block, scanner or camera, and pipette. The lateral flow assay uses a sample volume of 20 µL, requires only three steps over the course of 20 minutes, costs $0.80 per strip using commercial reagents, and performs consistently after short-term storage. By modifying the target capture, positive control, and probe sequences, the LFA may be adapted to detect other RNA targets. The LFA is capable of detecting short, amplified sequences or long, genomic sequences, although the LOD may increase with target size by an order of magnitude or more (data not shown). The LFA is suitable for low-resource settings and has the potential to be performed at the POC.

The LFA represents a different approach from other assays that may potentially serve as viral load tests. While other researchers have developed quantitative tests for p24 antigen to avoid the complications of nucleic acid tests [14], [15], the LFA detects HIV RNA, which is the traditional target for viral load tests. Like other lateral flow tests that use signal amplification to achieve appropriate sensitivity [12], [13], the LFA presented here uses gold enhancement to improve performance. However, because the LFA provides detection of nucleic acids after target amplification, the LFA may be used with any amplification method that generates RNA. For example, the LFA may be used in conjunction with microfluidic systems that have been designed to perform nucleic acid amplification [9], [10]. Therefore, the LFA may be integrated with new or existing amplification systems to measure HIV viral load.

Although the LFA performs well for detecting amplified HIV RNA, some of the required conditions may be difficult to achieve at the point-of-care. Currently, the LFA requires a heat block, which may be unavailable in low-resource settings because of cost and the requirement for electricity. To overcome these obstacles, the LFA may be used in conjunction with an inexpensive battery-powered heater or exothermal chemical heating unit based on engineered phase change materials [30], [31]. Another drawback of the LFA is that strips are left to dry before the data are collected, which requires additional time. In order to decrease the total assay time, strips may be dried quickly by heating or imaged while wet. In this study strips were imaged with a microscope, which often is not available in the field. To eliminate the necessity of expensive imaging equipment, strips may be imaged with a digital camera, point-of-care optical reader, or cell phone [32], [33]. Alternatively, the color of the detection zone may be compared to a color scale to completely eliminate the need for imaging equipment. Finally, the LFA requires multiple pipetting steps and a dilution series of standards to achieve quantitative results; other work has shown that multi-step assays may be integrated in a paper microfluidic device to simplify the procedure and avoid the need for a pipette [34]. Despite current limitations, the LFA may be modified or used in conjunction with other available technology to serve as an appropriate point-of-care test. Further work would be required to integrate the LFA with sample preparation and nucleic acid amplification in a format that is suitable for low resource settings.

We have described a quantitative LFA that detects amplified HIV RNA by using gold nanoparticle probes and gold enhancement. The assay has the potential to serve as part of a point-of-care test in low-resource settings. Because this assay serves as a detection platform, the LFA that may be adapted to quantify RNA targets for other diseases as well. The LFA may be integrated with amplification and sample preparation to comprise an HIV viral load test for low-resource settings. Point-of-care tests for viral load measurement in low resource settings have the potential to allow proper monitoring of HIV patients receiving ART, improving the management of HIV in the developing world.

## Supporting Information

Figure S1 **Detection of NASBA products in three additional experiments (A–C).** To the left, scanned images of LFA strips are shown at the appropriate dilution for the SBRs to fall within the linear range of the assay. The total number of *gag* RNA copies added to each NASBA reaction are shown next to each image. Note that although the contrast was adjusted for scanned images, raw images were used for signal-to-background calculations. To the right, the SBRs for the LFA strips are shown. ‘NTC’ = ‘no target control.’ An asterisk (*) denotes that 740 ng of total nucleic acid purified from lymphoblasts was added to the sample.
(TIF)

## References

[1] (2010) Global report: UNAIDS report on the global AIDS epidemic. Joint United Nations Programme on HIV/AIDS.

[2] (2009) Towards universal access: Scaling up priority HIV/AIDS interventions in the health sector. World Health Organization.

[3] Wang S, Xu F, Demirci U (2010) Advances in developing HIV-1 viral load assays for resource-limited settings. Biotechnology Advances: 770–781. 10.1016/j.biotechadv.2010.06.004 PMC2946488 20600784

[4] Volberding PA, Deeks SG (2010) Antiretroviral therapy and management of HIV infection. Lancet 376: 49–62. 20609987 10.1016/S0140-6736(10)60676-9

[5] Moore D, Awor A, Downing R, Kaplan J, Montaner J, et al (2008) CD4(+) T-Cell Count Monitoring Does Not Accurately Identify HIV-Infected Adults With Virologic Failure Receiving Antiretroviral Therapy. Jaids-Journal of Acquired Immune Deficiency Syndromes 49: 477–484. 10.1097/QAI.0b013e318186eb18 18989232

[6] Respess RA, Cachafeiro A, Withum D, Fiscus SA, Newman D, et al (2005) Evaluation of an ultrasensitive p24 antigen assay as a potential alternative to human immunodeficiency virus type 1 RNA viral load assay in resource-limited settings. Journal of Clinical Microbiology 43: 506–508. 15635029 10.1128/JCM.43.1.506-508.2005 PMC540096

[7] Stewart P, Cachafeiro A, Napravnik S, Eron JJ, Frank I, et al (2010) Performance characteristics of the Cavidi ExaVir viral load assay and the ultra-sensitive P24 assay relative to the Roche Monitor HIV-1 RNA assay. Journal of Clinical Virology 49: 198–204. 20832356 10.1016/j.jcv.2010.07.022 PMC3025774

[8] Fiscus S, Cheng B, Crowe S, Demeter L, Jennings C, et al.. (2006) HIV-1 viral load assays for resource-limited settings. Plos Medicine: 1743–1750. 10.1371/journal.pmed.0030417 PMC1592347 17032062

[9] Lee SH, Kim SW, Kang JY, Ahn CH (2008) A polymer lab-on-a-chip for reverse transcription (RT)-PCR based point-of-care clinical diagnostics. Lab on a Chip 8: 2121–2127. 19023475 10.1039/b811131f

[10] Dimov IK, Garcia-Cordero JL, O’Grady J, Poulsen CR, Viguier C, et al (2008) Integrated microfluidic tmRNA purification and real-time NASBA device for molecular diagnostics. Lab on a Chip 8: 2071–2078. 19023470 10.1039/b812515e

[11] Asiello P, Baeumner A (2011) Miniaturized isothermal nucleic acid amplification, a review. Lab on a Chip: 1420–1430. 10.1039/c0lc00666a 21387067

[12] Lee H, Dineva M, Chua Y, Ritchie A, Ushiro-Lumb I, et al (2010) Simple Amplification-Based Assay: A Nucleic Acid Based Point-of-Care Platform for HIV-1 Testing. Journal of Infectious Diseases 201: S65–S72. 20225949 10.1086/650385

[13] He Y, Zhang S, Zhang X, Baloda M, Gurung A, et al (2011) Ultrasensitive nucleic acid biosensor based on enzyme-gold nanoparticle dual label and lateral flow strip biosensor. Biosensors & Bioelectronics 26: 2018–2024. 20875950 10.1016/j.bios.2010.08.079

[14] Tang SX, Zhao JQ, Storhoff JJ, Norris PJ, Little RF, et al (2007) Nanoparticle-based biobarcode amplification assay (BCA) for sensitive and early detection of human immunodeficiency type 1 capsid (p24) antigen. Jaids-Journal of Acquired Immune Deficiency Syndromes 46: 231–237. 10.1097/QAI.0b013e31814a554b 17693896

[15] Parpia ZA, Elghanian R, Nabatiyan A, Hardie DR, Kelso DM (2010) p24 Antigen Rapid Test for Diagnosis of Acute Pediatric HIV Infection. Jaids-Journal of Acquired Immune Deficiency Syndromes 55: 413–419. 10.1097/QAI.0b013e3181f1afbc 20811289

[16] Martinez A, Phillips S, Whitesides G, Carrilho E (2010) Diagnostics for the Developing World: Microfluidic Paper-Based Analytical Devices. Analytical Chemistry: 3–10. 10.1021/ac9013989 20000334

[17] Fu E, Liang T, Houghtaling J, Ramachandran S, Ramsey S, et al (2011) Enhanced Sensitivity of Lateral Flow Tests Using a Two-Dimensional Paper Network Format. Analytical Chemistry 83: 7941–7946. 21936486 10.1021/ac201950g PMC3219509

[18] Carter DJ, Cary RB (2007) Lateral flow microarrays: a novel platform for rapid nucleic acid detection based on miniaturized lateral flow chromatography. Nucleic Acids Research 35. 10.1093/nar/gkm269 PMC1904290 17478499

[19] Martinez A, Phillips S, Nie Z, Cheng C, Carrilho E, et al (2010) Programmable diagnostic devices made from paper and tape. Lab on a Chip 10: 2499–2504. 20672179 10.1039/c0lc00021c

[20] Yu J, Ge L, Huang J, Wang S, Ge S (2011) Microfluidic paper-based chemiluminescence biosensor for simultaneous determination of glucose and uric acid. Lab on a Chip 11: 1286–1291. 21243159 10.1039/c0lc00524j

[21] Li X, Tian J, Shen W (2010) Quantitative biomarker assay with microfluidic paper-based analytical devices. Analytical and Bioanalytical Chemistry 396: 495–501. 19838826 10.1007/s00216-009-3195-9

[22] Fu E, Kauffman P, Lutz B, Yager P (2010) Chemical signal amplification in two-dimensional paper networks. Sensors and Actuators B-Chemical 149: 325–328. 10.1016/j.snb.2010.06.024 PMC2917776 20706615

[23] Gupta S, Huda S, Kilpatrick P, Velev O (2007) Characterization and optimization of gold nanoparticle-based silver-enhanced immunoassays. Analytical Chemistry 79: 3810–3820. 17429944 10.1021/ac062341m

[24] Thaxton C, Georganopoulou D, Mirkin C (2006) Gold nanoparticle probes for the detection of nucleic acid targets. Clinica Chimica Acta 363: 120–126. 10.1016/j.cccn.2005.05.042 16214124

[25] Hurst SJ, Lytton-Jean AKR, Mirkin CA (2006) Maximizing DNA loading on a range of gold nanoparticle sizes. Analytical Chemistry 78: 8313–8318. 17165821 10.1021/ac0613582 PMC2525614

[26] COMPTON J (1991) NUCLEIC-ACID SEQUENCE-BASED AMPLIFICATION. Nature 350: 91–92. 1706072 10.1038/350091a0

[27] Fields BN, Knipe DM, Howley PM (2007) Fields virology. Philadelphia: Wolters Kluwer Health/Lippincott Williams & Wilkins. 2 v. (xix, 3091, 3086 p.), [3094] p. of plates p.

[28] (2006) Guidelines for the Use of Anti-retroviral Agents in HIV-1-infected Adults and Adolescents. Panel on Antiretroviral Guidelines for Adults and Adolescents, Department of Health and Human Services.

[29] (2011) Guidelines for the Use of Anti-retroviral Agents in HIV-1-infected Adults and Adolescents. Panel on Antiretroviral Guidelines for Adults and Adolescents, Department of Health and Human Services.

[30] LaBarre P, Hawkins KR, Gerlach J, Wilmoth J, Beddoe A, et al (2011) A Simple, Inexpensive Device for Nucleic Acid Amplification without Electricity-Toward Instrument-Free Molecular Diagnostics in Low-Resource Settings. Plos One 6: 8. 10.1371/journal.pone.0019738 PMC3090398 21573065

[31] Liu C, Mauk MG, Hart R, Qiu X, Bau HH (2011) A self-heating cartridge for molecular diagnostics. Lab on a Chip 11: 2686–2692. 21734986 10.1039/c1lc20345b PMC12142467

[32] Ellerbee A, Phillips S, Siegel A, Mirica K, Martinez A, et al (2009) Quantifying Colorimetric Assays in Paper-Based Microfluidic Devices by Measuring the Transmission of Light through Paper. Analytical Chemistry 81: 8447–8452. 19722495 10.1021/ac901307q

[33] Zhu H, Yaglidere O, Su T, Tseng D, Ozcan A (2011) Cost-effective and compact wide-field fluorescent imaging on a cell-phone. Lab on a Chip 11: 315–322. 21063582 10.1039/c0lc00358a PMC3073081

[34] Fu E, Lutz B, Kauffman P, Yager P (2010) Controlled reagent transport in disposable 2D paper networks. Lab on a Chip 10: 918–920. 20300678 10.1039/b919614e PMC3228840

