# Supplementary figures and images for: A Lateral Flow Assay for Quantitative Detection of Amplified HIV-1 RNA
Source: PLoS One. 2012 Sep 21;7(9):e45611. doi: 10.1371/journal.pone.0045611 (PMC3448666; doi:10.1371/journal.pone.0045611)

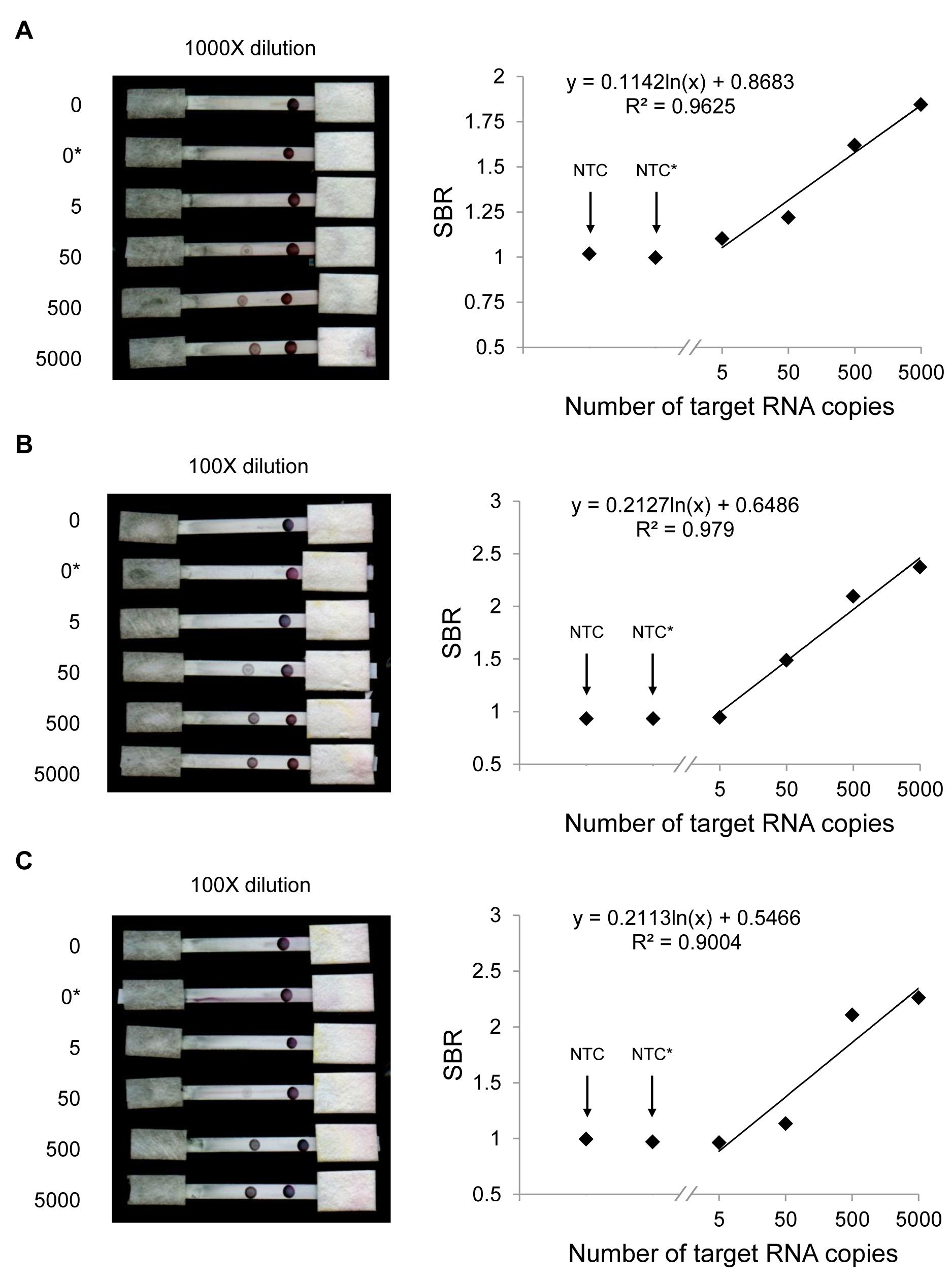

Supplement: Figure S1 — Detection of NASBA products in three additional experiments (A–C). To the left, scanned images of LFA strips are shown at the appropriate dilution for the SBRs to fall within the linear range of the assay. The total number of gag RNA copies added to each NASBA reaction are shown next to each image. Note that although the contrast was adjusted for scanned images, raw images were used for signal-to-background calculations. To the right, the SBRs for the LFA strips are shown. ‘NTC’ = ‘no target control.’ An asterisk (*) denotes that 740 ng of total nucleic acid purified from lymphoblasts was added to the sample. (TIF) [file pone.0045611.s001.tif]
